# 5,7,8,10,11,13,14,16-Octa­hydro-6,15-(ethanoxyethanoxyethano)-1,4:17,20-dietheno[9,12,6,15]benzodioxadiaza­cyclo­docosine^1^

**DOI:** 10.1107/S1600536812044017

**Published:** 2012-10-31

**Authors:** Heath A. Barnett, Frank R. Fronczek, Steven F. Watkins

**Affiliations:** a Department of Chemistry, Louisiana State University, Baton Rouge, LA 70803-1804, USA

## Abstract

The title compound, C_32_H_40_N_2_O_4_, is a 1,10-diaza-18-crown-6 cryptand with an *o*-terphenyl bridge. In the polyether ring, two adjacent –CH_2_– groups are disordered with very nearly equal populations of two conformers. The ordered bond lengths are normal, with average C—C = 1.511 (3) Å, C—O = 1.421 (3) Å, and C—N = 1.466 (4) Å. The r.m.s. deviations of the three rings of the terphenyl bridge vary from 0.007 to 0.009 Å and the two rings *ortho* to one another are twisted by 50.75 (5) and 47.76 (4)° with respect to the third ring. The N⋯N distance is 5.408 (1) Å.

## Related literature

For the synthesis of the title compound, see: Rossa & Vögtle (1981 ▶). For the structure of the NaSCN complex, see: Weber (1981 ▶). For a related structure, see: Vögtle *et al.* (1983 ▶). For the synthesis of cryptands, see: Dietrich *et al.* (1969*a* ▶,*b* ▶). For a background to guest–host inter­actions, see: Dunitz *et al.* (1974 ▶); Cram & Trueblood (1981 ▶); Cram (1988 ▶).
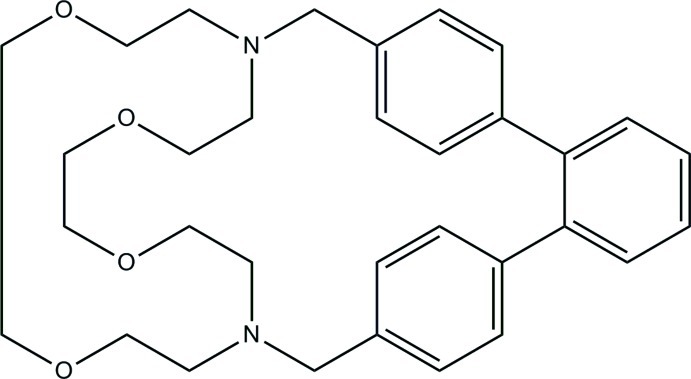


## Experimental

### 

#### Crystal data

C_32_H_40_N_2_O_4_
*M* *_r_* = 516.66
Triclinic, 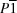

*a* = 9.6757 (3) Å
*b* = 12.1582 (4) Å
*c* = 12.5129 (5) Å
α = 88.178 (2)°
β = 82.616 (2)°
γ = 78.072 (2)°
*V* = 1428.26 (9) Å^3^
*Z* = 2
Mo *K*α radiation
μ = 0.08 mm^−1^
*T* = 100 K
0.33 × 0.32 × 0.22 mm

#### Data collection

Nonius KappaCCD diffractometer
Absorption correction: multi-scan (*SCALEPACK*; Otwinowski & Minor, 1997 ▶) *T*_min_ = 0.975, *T*_max_ = 0.983
10763 measured reflections
10763 independent reflections
5346 reflections with *I* > 2σ(*I*)

#### Refinement

*R*[*F* ^2^ > 2σ(*F* ^2^)] = 0.045
*wR*(*F* ^2^) = 0.115
*S* = 0.81
10763 reflections
351 parameters
H-atom parameters constrained
Δρ_max_ = 0.30 e Å^−3^
Δρ_min_ = −0.22 e Å^−3^

### 

Data collection: *COLLECT* (Nonius, 2000 ▶); cell refinement: *DENZO* and *SCALEPACK* (Otwinowski & Minor, 1997 ▶); data reduction: *DENZO* and *SCALEPACK*; program(s) used to solve structure: *SHELXS86* (Sheldrick, 2008 ▶); program(s) used to refine structure: *SHELXL97* (Sheldrick, 2008 ▶); molecular graphics: *ORTEP-3 for Windows* (Farrugia, 1997 ▶); software used to prepare material for publication: *WinGX* (Farrugia, 1999 ▶).

## Supplementary Material

Crystal structure: contains datablock(s) global, I. DOI: 10.1107/S1600536812044017/bv2212sup1.cif

Structure factors: contains datablock(s) I. DOI: 10.1107/S1600536812044017/bv2212Isup2.hkl

Supplementary material file. DOI: 10.1107/S1600536812044017/bv2212Isup3.cml

Additional supplementary materials:  crystallographic information; 3D view; checkCIF report

## Figures and Tables

**Fig. 1.**
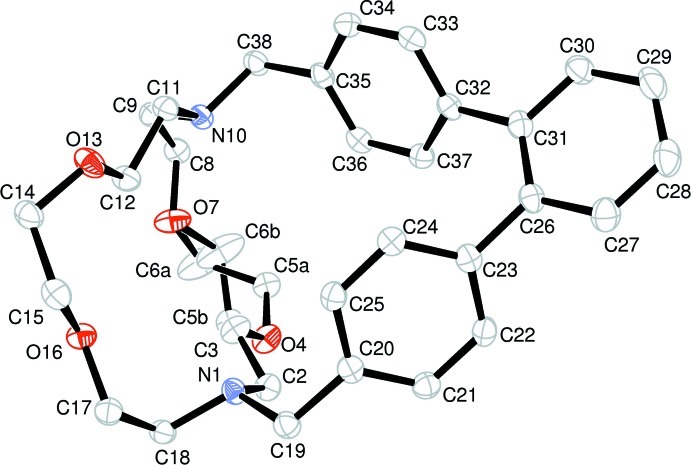
View of (I) (50% probability displacement ellipsoids)

## supplementary crystallographic information

### Comment

Cryptands, first synthesized in Lehn's laboratories (Dietrich *et al.* 1969*a*,*b*) have been designed for many years as supramolecular hosts for complexation of metal ions and other guests (Cram, 1988). Dunitz *et al.* (1974) and Cram & Trueblood (1981) emphasized the cavity size of the host matching the size of the guest, both by construction of the host and by its organization by the guest. In the title compound, C_32_H_40_N_2_O_4_, an *o*-terphenyl bridge was inserted into the macrobicyclic framework by Rossa & Vögtle (1981) to control the cavity size of cryptand-[2.2.2]. We report here the structure of the altered cryptand.

The molecule has approximate *C*_2_ symmetry, with the central ring of the terphenyl group forming dihedral angles of 50.75 (5) and 47.76 (4)° with the other two benzene rings. The three rings of the terphenyl bridge are essentially planar, with r.m.s. deviations ranging from 0.007 to 0.009 Å. In the diaza-18-crown-6 ring, one –(CH_2_)_2_– group (C5 and C6) is disordered with very nearly equal populations of the two conformers [0.490 (3) and 0.510 (3)]. The bond lengths in the ordered part of the molecule are normal, with average C—C = 1.511 (3) Å, C—O = 1.421 (3) Å, and C—N = 1.466 (4) Å.

The Na^+^ complex of the title cryptand has been reported (Weber, 1981) as the thiocyanate salt, methanol solvate. In that structure, the conformation of the terphenyl subunit is quite similar to that in the title cryptand, with the central phenyl group forming dihedral angles of 49.2 and 54.9° with the other two. The N···N distance in the Na^+^ complex, 5.341 (1) Å, is also not much different from that in the uncomplexed cryptand, 5.408 (1) Å.

### Experimental

The title compound was prepared as decribed by Rossa & Vögtle (1981), and the sample was kindly provided by Professor Vögtle. Crystals were grown from chloroform.

### Refinement

All H atoms were placed in calculated positions, guided by difference maps. The C—H bond distances were restrained to the range 0.95 to 0.99 Å, with *U*_iso_=1.2*U*_eq_ and thereafter refined as riding.

Two carbon atoms, C5 and C6, and their attached hydrogen atoms, are disordered and were treated as separately attached groups (A and B) using the PART command in *SHELXL97* (Sheldrick, 2008). Their occupation factors were refined as parameter *x* and 1 - *x*, with *x* = 0.490 (3).

### Crystal data

**Table:**

C_32_H_40_N_2_O_4_	*Z* = 2
*M**_r_* = 516.66	*F*(000) = 556
Triclinic, *P*1	*D*_x_ = 1.201 Mg m^−^^3^
Hall symbol: -P 1	Mo *K*α radiation, λ = 0.71073 Å
*a* = 9.6757 (3) Å	Cell parameters from 10814 reflections
*b* = 12.1582 (4) Å	θ = 2.6–33.1°
*c* = 12.5129 (5) Å	µ = 0.08 mm^−^^1^
α = 88.178 (2)°	*T* = 100 K
β = 82.616 (2)°	Fragment, colorless
γ = 78.072 (2)°	0.33 × 0.32 × 0.22 mm
*V* = 1428.26 (9) Å^3^	

### Data collection

**Table:**

Nonius KappaCCD diffractometer	10763 independent reflections
Radiation source: sealed tube	5346 reflections with *I* > 2σ(*I*)
Graphite monochromator	*R*_int_ = 0
Detector resolution: 9 pixels mm^-1^	θ_max_ = 33.1°, θ_min_ = 2.6°
ω and φ scans	*h* = −14→14
Absorption correction: multi-scan (*SCALEPACK*; Otwinowski & Minor, 1997)	*k* = −18→18
*T*_min_ = 0.975, *T*_max_ = 0.983	*l* = 0→19
10763 measured reflections	

### Refinement

**Table:**

Refinement on *F*^2^	Secondary atom site location: difference Fourier map
Least-squares matrix: full	Hydrogen site location: inferred from neighbouring sites
*R*[*F*^2^ > 2σ(*F*^2^)] = 0.045	H-atom parameters constrained
*wR*(*F*^2^) = 0.115	*w* = 1/[σ^2^(*F*_o_^2^) + (0.0599*P*)^2^ + 0.0637*P*] where *P* = (*F*_o_^2^ + 2*F*_c_^2^)/3
*S* = 0.81	(Δ/σ)_max_ = 0.001
10763 reflections	Δρ_max_ = 0.30 e Å^−^^3^
351 parameters	Δρ_min_ = −0.22 e Å^−^^3^
0 restraints	Extinction correction: *SHELXL97* (Sheldrick, 2008), Fc^*^=kFc[1+0.001xFc^2^λ^3^/sin(2θ)]^-1/4^
0 constraints	Extinction coefficient: 0.0078 (12)
Primary atom site location: structure-invariant direct methods	

### Special details

**Table:**

Geometry. All e.s.d.'s (except the e.s.d. in the dihedral angle between two l.s. planes) are estimated using the full covariance matrix. The cell e.s.d.'s are taken into account individually in the estimation of e.s.d.'s in distances, angles and torsion angles; correlations between e.s.d.'s in cell parameters are only used when they are defined by crystal symmetry. An approximate (isotropic) treatment of cell e.s.d.'s is used for estimating e.s.d.'s involving l.s. planes.

### Fractional atomic coordinates and isotropic or equivalent isotropic displacement parameters (Å^2^)

**Table:**

	*x*	*y*	*z*	*U*_iso_*/*U*_eq_	Occ. (<1)
N1	−0.23215 (9)	0.53384 (7)	0.19296 (7)	0.0211 (2)	
C2	−0.37788 (11)	0.58008 (10)	0.24093 (9)	0.0247 (2)	
H2A	−0.4347	0.5208	0.2433	0.03*	
H2B	−0.421	0.642	0.1947	0.03*	
C3	−0.38478 (11)	0.62427 (10)	0.35394 (9)	0.0270 (3)	
H3A	−0.3355	0.5654	0.4003	0.032*	
H3B	−0.3393	0.6902	0.3524	0.032*	
O4	−0.53150 (8)	0.65506 (7)	0.39325 (6)	0.02816 (19)	
C5A	−0.5774 (2)	0.7325 (2)	0.47940 (19)	0.0250 (7)	0.490 (3)
H5A1	−0.6827	0.7488	0.4907	0.03*	0.490 (3)
H5A2	−0.5473	0.8037	0.4569	0.03*	0.490 (3)
C6A	−0.5257 (3)	0.6977 (4)	0.5841 (3)	0.0393 (7)	0.490 (3)
H6AA	−0.5853	0.7448	0.6426	0.047*	0.490 (3)
H6AB	−0.5312	0.6182	0.5996	0.047*	0.490 (3)
C5B	−0.5732 (2)	0.6471 (2)	0.50314 (18)	0.0268 (7)	0.510 (3)
H5B1	−0.5277	0.5718	0.528	0.032*	0.510 (3)
H5B2	−0.6774	0.6517	0.5144	0.032*	0.510 (3)
C6B	−0.54004 (7)	0.73216 (6)	0.57237 (5)	0.0393 (7)	0.510 (3)
H6BA	−0.5715	0.8083	0.5424	0.047*	0.510 (3)
H6BB	−0.5917	0.7289	0.6456	0.047*	0.510 (3)
O7	−0.38556 (7)	0.71046 (6)	0.57801 (5)	0.0337 (2)	
C8	−0.35602 (7)	0.80673 (6)	0.62382 (5)	0.0246 (2)	
H8A	−0.3743	0.803	0.7034	0.03*	
H8B	−0.418	0.8755	0.5989	0.03*	
C9	−0.20174 (11)	0.80985 (9)	0.58898 (8)	0.0224 (2)	
H9A	−0.172	0.8631	0.6352	0.027*	
H9B	−0.1426	0.7344	0.5992	0.027*	
N10	−0.17621 (9)	0.84405 (7)	0.47596 (7)	0.02067 (19)	
C11	−0.02881 (11)	0.80186 (9)	0.42935 (9)	0.0227 (2)	
H11A	0.0359	0.8144	0.4809	0.027*	
H11B	−0.0079	0.8449	0.3628	0.027*	
C12	0.00009 (11)	0.67766 (9)	0.40272 (9)	0.0239 (2)	
H12A	−0.0193	0.6329	0.4683	0.029*	
H12B	−0.0607	0.6636	0.3489	0.029*	
O13	0.14635 (8)	0.64881 (6)	0.36029 (7)	0.02792 (19)	
C14	0.20675 (12)	0.53217 (9)	0.35947 (10)	0.0274 (3)	
H14A	0.1765	0.4994	0.4299	0.033*	
H14B	0.3117	0.5222	0.3515	0.033*	
C15	0.16638 (11)	0.46794 (9)	0.27126 (9)	0.0259 (2)	
H15A	0.1703	0.5114	0.2031	0.031*	
H15B	0.2344	0.3952	0.2598	0.031*	
O16	0.02643 (7)	0.44884 (6)	0.30085 (6)	0.02359 (18)	
C17	−0.02009 (12)	0.38896 (10)	0.22075 (9)	0.0268 (3)	
H17A	0.0121	0.307	0.2309	0.032*	
H17B	0.0205	0.4097	0.148	0.032*	
C18	−0.18012 (12)	0.41909 (9)	0.23150 (9)	0.0235 (2)	
H18A	−0.2153	0.3652	0.1896	0.028*	
H18B	−0.2188	0.4126	0.3081	0.028*	
C19	−0.22206 (12)	0.53917 (9)	0.07518 (9)	0.0237 (2)	
H19A	−0.3009	0.5094	0.0513	0.028*	
H19B	−0.1312	0.4913	0.0438	0.028*	
C20	−0.22940 (11)	0.65830 (9)	0.03457 (8)	0.0217 (2)	
C21	−0.33622 (11)	0.71247 (9)	−0.02373 (9)	0.0240 (2)	
H21	−0.4051	0.673	−0.0418	0.029*	
C22	−0.34428 (11)	0.82403 (9)	−0.05634 (8)	0.0237 (2)	
H22	−0.4183	0.8595	−0.0965	0.028*	
C23	−0.24558 (11)	0.88407 (9)	−0.03088 (8)	0.0216 (2)	
C24	−0.13529 (11)	0.82843 (9)	0.02581 (9)	0.0224 (2)	
H24	−0.0648	0.8671	0.0421	0.027*	
C25	−0.12808 (11)	0.71798 (9)	0.05823 (8)	0.0225 (2)	
H25	−0.0531	0.6819	0.0972	0.027*	
C26	−0.25954 (11)	1.00549 (9)	−0.06088 (9)	0.0224 (2)	
C27	−0.27508 (12)	1.03916 (10)	−0.16710 (9)	0.0293 (3)	
H27	−0.2808	0.9846	−0.2182	0.035*	
C28	−0.28245 (13)	1.15045 (11)	−0.20031 (10)	0.0329 (3)	
H28	−0.293	1.1714	−0.2731	0.039*	
C29	−0.27428 (12)	1.23011 (10)	−0.12599 (10)	0.0321 (3)	
H29	−0.277	1.306	−0.1478	0.039*	
C30	−0.26221 (12)	1.19882 (10)	−0.01984 (10)	0.0279 (3)	
H30	−0.2581	1.2544	0.0306	0.033*	
C31	−0.25585 (11)	1.08767 (9)	0.01550 (9)	0.0221 (2)	
C32	−0.24813 (11)	1.06006 (9)	0.13170 (9)	0.0213 (2)	
C33	−0.15041 (11)	1.09725 (9)	0.18699 (9)	0.0241 (2)	
H33	−0.0899	1.1427	0.1508	0.029*	
C34	−0.14044 (12)	1.06865 (9)	0.29445 (9)	0.0244 (2)	
H34	−0.0729	1.0948	0.3306	0.029*	
C35	−0.22686 (11)	1.00284 (9)	0.34998 (9)	0.0222 (2)	
C36	−0.32687 (11)	0.96731 (9)	0.29568 (9)	0.0229 (2)	
H36	−0.3883	0.923	0.3326	0.027*	
C37	−0.33787 (11)	0.99569 (9)	0.18866 (9)	0.0216 (2)	
H37	−0.4073	0.9712	0.1534	0.026*	
C38	−0.21402 (12)	0.96682 (9)	0.46525 (9)	0.0244 (2)	
H38A	−0.1403	1.0004	0.4921	0.029*	
H38B	−0.3057	0.9953	0.5102	0.029*	

### Atomic displacement parameters (Å^2^)

**Table:**

	*U*^11^	*U*^22^	*U*^33^	*U*^12^	*U*^13^	*U*^23^
N1	0.0243 (5)	0.0195 (5)	0.0185 (5)	−0.0034 (4)	−0.0008 (4)	0.0012 (4)
C2	0.0226 (5)	0.0265 (6)	0.0246 (6)	−0.0044 (5)	−0.0022 (4)	−0.0001 (5)
C3	0.0194 (5)	0.0364 (7)	0.0251 (6)	−0.0069 (5)	0.0009 (4)	−0.0047 (5)
O4	0.0193 (4)	0.0385 (5)	0.0254 (4)	−0.0042 (3)	0.0005 (3)	−0.0042 (4)
C5A	0.0188 (11)	0.0283 (16)	0.0270 (13)	−0.0040 (10)	0.0011 (9)	−0.0065 (11)
C6A	0.0244 (8)	0.063 (2)	0.0326 (9)	−0.0174 (10)	0.0067 (6)	−0.0113 (11)
C5B	0.0221 (11)	0.0331 (16)	0.0250 (12)	−0.0092 (10)	0.0027 (9)	0.0036 (10)
C6B	0.0244 (8)	0.063 (2)	0.0326 (9)	−0.0174 (10)	0.0067 (6)	−0.0113 (11)
O7	0.0271 (4)	0.0398 (5)	0.0357 (5)	−0.0136 (4)	0.0049 (4)	−0.0161 (4)
C8	0.0291 (6)	0.0230 (6)	0.0196 (6)	−0.0025 (5)	0.0010 (4)	−0.0034 (4)
C9	0.0272 (6)	0.0219 (6)	0.0184 (5)	−0.0043 (4)	−0.0045 (4)	−0.0006 (4)
N10	0.0240 (5)	0.0177 (4)	0.0193 (5)	−0.0026 (4)	−0.0017 (4)	−0.0001 (4)
C11	0.0226 (5)	0.0200 (5)	0.0257 (6)	−0.0059 (4)	−0.0016 (4)	0.0000 (4)
C12	0.0200 (5)	0.0216 (6)	0.0297 (6)	−0.0051 (4)	0.0002 (4)	−0.0023 (5)
O13	0.0200 (4)	0.0189 (4)	0.0424 (5)	−0.0028 (3)	0.0030 (3)	0.0011 (3)
C14	0.0196 (5)	0.0204 (6)	0.0411 (7)	−0.0020 (5)	−0.0033 (5)	−0.0007 (5)
C15	0.0222 (5)	0.0235 (6)	0.0298 (6)	−0.0029 (5)	0.0022 (5)	0.0013 (5)
O16	0.0230 (4)	0.0249 (4)	0.0235 (4)	−0.0064 (3)	−0.0016 (3)	−0.0037 (3)
C17	0.0315 (6)	0.0226 (6)	0.0253 (6)	−0.0008 (5)	−0.0065 (5)	−0.0045 (5)
C18	0.0298 (6)	0.0196 (5)	0.0227 (6)	−0.0077 (5)	−0.0051 (5)	0.0000 (4)
C19	0.0274 (6)	0.0214 (6)	0.0215 (6)	−0.0037 (5)	−0.0018 (4)	−0.0012 (4)
C20	0.0249 (5)	0.0220 (6)	0.0156 (5)	−0.0018 (4)	0.0021 (4)	−0.0019 (4)
C21	0.0252 (6)	0.0254 (6)	0.0216 (6)	−0.0059 (5)	−0.0022 (4)	−0.0020 (5)
C22	0.0249 (6)	0.0252 (6)	0.0196 (6)	−0.0013 (5)	−0.0048 (4)	0.0013 (4)
C23	0.0227 (5)	0.0221 (6)	0.0180 (5)	−0.0022 (4)	0.0015 (4)	0.0000 (4)
C24	0.0203 (5)	0.0245 (6)	0.0214 (6)	−0.0038 (4)	0.0006 (4)	−0.0014 (4)
C25	0.0210 (5)	0.0241 (6)	0.0204 (6)	0.0001 (4)	−0.0018 (4)	−0.0011 (4)
C26	0.0202 (5)	0.0232 (6)	0.0224 (6)	−0.0033 (4)	−0.0004 (4)	0.0035 (4)
C27	0.0316 (6)	0.0320 (7)	0.0230 (6)	−0.0050 (5)	−0.0021 (5)	0.0023 (5)
C28	0.0367 (7)	0.0373 (7)	0.0244 (6)	−0.0082 (6)	−0.0041 (5)	0.0114 (5)
C29	0.0341 (7)	0.0266 (6)	0.0365 (7)	−0.0093 (5)	−0.0054 (5)	0.0127 (5)
C30	0.0286 (6)	0.0253 (6)	0.0310 (7)	−0.0090 (5)	−0.0040 (5)	0.0047 (5)
C31	0.0188 (5)	0.0231 (6)	0.0237 (6)	−0.0042 (4)	−0.0012 (4)	0.0039 (4)
C32	0.0215 (5)	0.0171 (5)	0.0231 (6)	−0.0009 (4)	0.0003 (4)	0.0005 (4)
C33	0.0263 (6)	0.0193 (5)	0.0275 (6)	−0.0080 (5)	−0.0010 (5)	0.0013 (4)
C34	0.0265 (6)	0.0217 (6)	0.0260 (6)	−0.0062 (5)	−0.0048 (5)	−0.0022 (5)
C35	0.0266 (6)	0.0163 (5)	0.0214 (6)	−0.0006 (4)	−0.0005 (4)	−0.0017 (4)
C36	0.0238 (5)	0.0186 (5)	0.0245 (6)	−0.0036 (4)	0.0022 (4)	0.0005 (4)
C37	0.0198 (5)	0.0204 (5)	0.0239 (6)	−0.0033 (4)	−0.0015 (4)	−0.0014 (4)
C38	0.0307 (6)	0.0183 (5)	0.0235 (6)	−0.0033 (5)	−0.0035 (5)	−0.0016 (4)

### Geometric parameters (Å, º)

**Table:**

N1—C2	1.4632 (14)	O16—C17	1.4265 (13)
N1—C19	1.4647 (15)	C17—C18	1.5050 (16)
N1—C18	1.4703 (14)	C17—H17A	0.99
C2—C3	1.5173 (16)	C17—H17B	0.99
C2—H2A	0.99	C18—H18A	0.99
C2—H2B	0.99	C18—H18B	0.99
C3—O4	1.4178 (13)	C19—C20	1.5104 (16)
C3—H3A	0.99	C19—H19A	0.99
C3—H3B	0.99	C19—H19B	0.99
O4—C5B	1.389 (2)	C20—C21	1.3842 (16)
O4—C5A	1.419 (2)	C20—C25	1.3992 (15)
C5A—C6A	1.480 (5)	C21—C22	1.3934 (16)
C5A—H5A1	0.99	C21—H21	0.95
C5A—H5A2	0.99	C22—C23	1.3884 (16)
C6A—O7	1.389 (3)	C22—H22	0.95
C6A—H6AA	0.99	C23—C24	1.4018 (15)
C6A—H6AB	0.99	C23—C26	1.4945 (16)
C5B—C6B	1.479 (2)	C24—C25	1.3808 (16)
C5B—H5B1	0.99	C24—H24	0.95
C5B—H5B2	0.99	C25—H25	0.95
C6B—O7	1.4737	C26—C27	1.3957 (16)
C6B—H6BA	0.99	C26—C31	1.4133 (15)
C6B—H6BB	0.99	C27—C28	1.3930 (17)
O7—C8	1.4153	C27—H27	0.95
C8—C9	1.5084 (13)	C28—C29	1.3845 (17)
C8—H8A	0.99	C28—H28	0.95
C8—H8B	0.99	C29—C30	1.3827 (17)
C9—N10	1.4660 (15)	C29—H29	0.95
C9—H9A	0.99	C30—C31	1.4001 (16)
C9—H9B	0.99	C30—H30	0.95
N10—C11	1.4630 (14)	C31—C32	1.4896 (16)
N10—C38	1.4677 (14)	C32—C33	1.3935 (15)
C11—C12	1.5175 (15)	C32—C37	1.4003 (15)
C11—H11A	0.99	C33—C34	1.3885 (16)
C11—H11B	0.99	C33—H33	0.95
C12—O13	1.4217 (13)	C34—C35	1.3836 (16)
C12—H12A	0.99	C34—H34	0.95
C12—H12B	0.99	C35—C36	1.3947 (15)
O13—C14	1.4176 (14)	C35—C38	1.5069 (16)
C14—C15	1.5074 (16)	C36—C37	1.3842 (16)
C14—H14A	0.99	C36—H36	0.95
C14—H14B	0.99	C37—H37	0.95
C15—O16	1.4215 (13)	C38—H38A	0.99
C15—H15A	0.99	C38—H38B	0.99
C15—H15B	0.99		
			
C2—N1—C19	110.31 (8)	C14—C15—H15B	109.8
C2—N1—C18	112.01 (9)	H15A—C15—H15B	108.2
C19—N1—C18	111.70 (8)	C15—O16—C17	112.76 (9)
N1—C2—C3	112.64 (9)	O16—C17—C18	108.22 (9)
N1—C2—H2A	109.1	O16—C17—H17A	110.1
C3—C2—H2A	109.1	C18—C17—H17A	110.1
N1—C2—H2B	109.1	O16—C17—H17B	110.1
C3—C2—H2B	109.1	C18—C17—H17B	110.1
H2A—C2—H2B	107.8	H17A—C17—H17B	108.4
O4—C3—C2	105.97 (9)	N1—C18—C17	111.99 (9)
O4—C3—H3A	110.5	N1—C18—H18A	109.2
C2—C3—H3A	110.5	C17—C18—H18A	109.2
O4—C3—H3B	110.5	N1—C18—H18B	109.2
C2—C3—H3B	110.5	C17—C18—H18B	109.2
H3A—C3—H3B	108.7	H18A—C18—H18B	107.9
C5B—O4—C3	118.81 (12)	N1—C19—C20	111.27 (8)
C3—O4—C5A	119.96 (11)	N1—C19—H19A	109.4
O4—C5A—C6A	116.8 (3)	C20—C19—H19A	109.4
O4—C5A—H5A1	108.1	N1—C19—H19B	109.4
C6A—C5A—H5A1	108.1	C20—C19—H19B	109.4
O4—C5A—H5A2	108.1	H19A—C19—H19B	108
C6A—C5A—H5A2	108.1	C21—C20—C25	118.19 (10)
H5A1—C5A—H5A2	107.3	C21—C20—C19	122.03 (10)
O7—C6A—C5A	108.2 (3)	C25—C20—C19	119.76 (10)
O7—C6A—H6AA	110.1	C20—C21—C22	120.98 (10)
C5A—C6A—H6AA	110.1	C20—C21—H21	119.5
O7—C6A—H6AB	110.1	C22—C21—H21	119.5
C5A—C6A—H6AB	110.1	C23—C22—C21	120.87 (10)
H6AA—C6A—H6AB	108.4	C23—C22—H22	119.6
O4—C5B—C6B	116.73 (18)	C21—C22—H22	119.6
O4—C5B—H5B1	108.1	C22—C23—C24	118.18 (10)
C6B—C5B—H5B1	108.1	C22—C23—C26	120.55 (10)
O4—C5B—H5B2	108.1	C24—C23—C26	121.25 (10)
C6B—C5B—H5B2	108.1	C25—C24—C23	120.68 (10)
H5B1—C5B—H5B2	107.3	C25—C24—H24	119.7
O7—C6B—C5B	109.69 (9)	C23—C24—H24	119.7
O7—C6B—H6BA	109.7	C24—C25—C20	121.07 (10)
C5B—C6B—H6BA	109.7	C24—C25—H25	119.5
O7—C6B—H6BB	109.7	C20—C25—H25	119.5
C5B—C6B—H6BB	109.7	C27—C26—C31	118.76 (10)
H6BA—C6B—H6BB	108.2	C27—C26—C23	119.57 (10)
C6A—O7—C8	119.25 (18)	C31—C26—C23	121.67 (10)
C8—O7—C6B	107.2	C28—C27—C26	121.87 (11)
O7—C8—C9	108.1	C28—C27—H27	119.1
O7—C8—H8A	110.1	C26—C27—H27	119.1
C9—C8—H8A	110.1	C29—C28—C27	119.19 (11)
O7—C8—H8B	110.1	C29—C28—H28	120.4
C9—C8—H8B	110.1	C27—C28—H28	120.4
H8A—C8—H8B	108.4	C30—C29—C28	119.76 (12)
N10—C9—C8	112.30 (8)	C30—C29—H29	120.1
N10—C9—H9A	109.1	C28—C29—H29	120.1
C8—C9—H9A	109.1	C29—C30—C31	122.00 (11)
N10—C9—H9B	109.1	C29—C30—H30	119
C8—C9—H9B	109.1	C31—C30—H30	119
H9A—C9—H9B	107.9	C30—C31—C26	118.36 (10)
C11—N10—C9	111.85 (8)	C30—C31—C32	119.43 (10)
C11—N10—C38	110.53 (9)	C26—C31—C32	122.19 (10)
C9—N10—C38	110.63 (8)	C33—C32—C37	117.92 (10)
N10—C11—C12	112.35 (9)	C33—C32—C31	120.80 (10)
N10—C11—H11A	109.1	C37—C32—C31	121.29 (10)
C12—C11—H11A	109.1	C34—C33—C32	120.74 (11)
N10—C11—H11B	109.1	C34—C33—H33	119.6
C12—C11—H11B	109.1	C32—C33—H33	119.6
H11A—C11—H11B	107.9	C35—C34—C33	121.31 (10)
O13—C12—C11	105.86 (9)	C35—C34—H34	119.3
O13—C12—H12A	110.6	C33—C34—H34	119.3
C11—C12—H12A	110.6	C34—C35—C36	118.20 (11)
O13—C12—H12B	110.6	C34—C35—C38	122.29 (10)
C11—C12—H12B	110.6	C36—C35—C38	119.50 (10)
H12A—C12—H12B	108.7	C37—C36—C35	120.89 (11)
C14—O13—C12	115.10 (9)	C37—C36—H36	119.6
O13—C14—C15	114.27 (10)	C35—C36—H36	119.6
O13—C14—H14A	108.7	C36—C37—C32	120.92 (10)
C15—C14—H14A	108.7	C36—C37—H37	119.5
O13—C14—H14B	108.7	C32—C37—H37	119.5
C15—C14—H14B	108.7	N10—C38—C35	111.78 (8)
H14A—C14—H14B	107.6	N10—C38—H38A	109.3
O16—C15—C14	109.39 (9)	C35—C38—H38A	109.3
O16—C15—H15A	109.8	N10—C38—H38B	109.3
C14—C15—H15A	109.8	C35—C38—H38B	109.3
O16—C15—H15B	109.8	H38A—C38—H38B	107.9
			
C19—N1—C2—C3	−154.83 (10)	C21—C22—C23—C26	−176.63 (10)
C18—N1—C2—C3	80.09 (12)	C22—C23—C24—C25	−2.00 (15)
N1—C2—C3—O4	−174.14 (9)	C26—C23—C24—C25	176.41 (9)
C2—C3—O4—C5B	149.61 (15)	C23—C24—C25—C20	0.65 (16)
C2—C3—O4—C5A	−158.83 (15)	C21—C20—C25—C24	0.93 (15)
C5B—O4—C5A—C6A	38.8 (2)	C19—C20—C25—C24	−177.45 (9)
C3—O4—C5A—C6A	−63.2 (3)	C22—C23—C26—C27	−52.09 (14)
O4—C5A—C6A—O7	77.2 (4)	C24—C23—C26—C27	129.53 (11)
C3—O4—C5B—C6B	72.7 (2)	C22—C23—C26—C31	128.64 (11)
C5A—O4—C5B—C6B	−31.95 (16)	C24—C23—C26—C31	−49.74 (15)
O4—C5B—C6B—O7	−71.37 (18)	C31—C26—C27—C28	2.06 (16)
C5A—C6A—O7—C8	100.0 (2)	C23—C26—C27—C28	−177.23 (10)
C5A—C6A—O7—C6B	51.1 (4)	C26—C27—C28—C29	−0.05 (18)
C5B—C6B—O7—C6A	−56.4 (4)	C27—C28—C29—C30	−1.45 (18)
C5B—C6B—O7—C8	167.06 (11)	C28—C29—C30—C31	0.92 (18)
C6A—O7—C8—C9	−170.1 (2)	C29—C30—C31—C26	1.10 (16)
C6B—O7—C8—C9	−156.45 (5)	C29—C30—C31—C32	−177.80 (10)
O7—C8—C9—N10	74.35 (8)	C27—C26—C31—C30	−2.53 (15)
C8—C9—N10—C11	−155.18 (8)	C23—C26—C31—C30	176.75 (10)
C8—C9—N10—C38	81.14 (10)	C27—C26—C31—C32	176.33 (10)
C9—N10—C11—C12	76.27 (11)	C23—C26—C31—C32	−4.39 (15)
C38—N10—C11—C12	−159.99 (9)	C30—C31—C32—C33	−48.86 (14)
N10—C11—C12—O13	−179.07 (8)	C26—C31—C32—C33	132.28 (11)
C11—C12—O13—C14	162.24 (9)	C30—C31—C32—C37	131.47 (11)
C12—O13—C14—C15	76.42 (13)	C26—C31—C32—C37	−47.38 (14)
O13—C14—C15—O16	−78.05 (12)	C37—C32—C33—C34	1.60 (15)
C14—C15—O16—C17	179.56 (9)	C31—C32—C33—C34	−178.07 (10)
C15—O16—C17—C18	−153.77 (9)	C32—C33—C34—C35	−0.13 (16)
C2—N1—C18—C17	−156.80 (9)	C33—C34—C35—C36	−1.17 (16)
C19—N1—C18—C17	78.88 (11)	C33—C34—C35—C38	177.48 (10)
O16—C17—C18—N1	72.81 (12)	C34—C35—C36—C37	0.97 (15)
C2—N1—C19—C20	73.08 (11)	C38—C35—C36—C37	−177.73 (9)
C18—N1—C19—C20	−161.66 (9)	C35—C36—C37—C32	0.53 (15)
N1—C19—C20—C21	−119.16 (11)	C33—C32—C37—C36	−1.80 (15)
N1—C19—C20—C25	59.15 (13)	C31—C32—C37—C36	177.87 (9)
C25—C20—C21—C22	−1.15 (15)	C11—N10—C38—C35	70.84 (11)
C19—C20—C21—C22	177.19 (10)	C9—N10—C38—C35	−164.72 (9)
C20—C21—C22—C23	−0.23 (16)	C34—C35—C38—N10	−118.41 (11)
C21—C22—C23—C24	1.79 (15)	C36—C35—C38—N10	60.23 (13)
